# Supplementation with a Specific Combination of Metabolic Cofactors Ameliorates Non-Alcoholic Fatty Liver Disease, Hepatic Fibrosis, and Insulin Resistance in Mice

**DOI:** 10.3390/nu13103532

**Published:** 2021-10-09

**Authors:** Sergio Quesada-Vázquez, Marina Colom-Pellicer, Èlia Navarro-Masip, Gerard Aragonès, Josep M. Del Bas, Antoni Caimari, Xavier Escoté

**Affiliations:** 1 Eurecat, Technology Centre of Catalunya, Nutrition and Health Unit, 43204 Reus, Spain; sergio.quesada@eurecat.org (S.Q.-V.); josep.delbas@eurecat.org (J.M.D.B.); 2 Nutrigenomics Research Group, Department of Biochemistry and Biotechnology, Universitat Rovira i Virgili, 43007 Tarragona, Spain; marina.colom@urv.cat (M.C.-P.); elia.navarro@urv.cat (È.N.-M.); gerard.aragones@urv.cat (G.A.); 3 Eurecat, Centre Tecnològic de Catalunya, Biotechnology Area, 43204 Reus, Spain; antoni.caimari@eurecat.org

**Keywords:** NAFLD, liver disease, steatosis, hepatic inflammation, hepatic insulin resistance

## Abstract

Non-alcoholic fatty liver disease (NAFLD) and non-alcoholic steatohepatitis (NASH) have emerged as the leading causes of chronic liver disease in the world. Obesity, insulin resistance, and dyslipidemia are multifactorial risk factors strongly associated with NAFLD/NASH. Here, a specific combination of metabolic cofactors (a multi-ingredient; MI) containing precursors of glutathione (GSH) and nicotinamide adenine dinucleotide (NAD^+^) (betaine, N-acetyl-cysteine, L-carnitine and nicotinamide riboside) was evaluated as effective treatment for the NAFLD/NASH pathophysiology. Six-week-old male mice were randomly divided into control diet animals and animals exposed to a high fat and high fructose/sucrose diet to induce NAFLD. After 16 weeks, diet-induced NAFLD mice were distributed into two groups, treated with the vehicle (HFHFr group) or with a combination of metabolic cofactors (MI group) for 4 additional weeks, and blood and liver were obtained from all animals for biochemical, histological, and molecular analysis. The MI treatment reduced liver steatosis, decreasing liver weight and hepatic lipid content, and liver injury, as evidenced by a pronounced decrease in serum levels of liver transaminases. Moreover, animals supplemented with the MI cocktail showed a reduction in the gene expression of some proinflammatory cytokines when compared with their HFHFr counterparts. In addition, MI supplementation was effective in decreasing hepatic fibrosis and improving insulin sensitivity, as observed by histological analysis, as well as a reduction in fibrotic gene expression (*Col1α1*) and improved Akt activation, respectively. Taken together, supplementation with this specific combination of metabolic cofactors ameliorates several features of NAFLD, highlighting this treatment as a potential efficient therapy against this disease in humans.

## 1. Introduction

Non-alcoholic fatty liver disease (NAFLD) and non-alcoholic steatohepatitis (NASH) have emerged as the leading causes of chronic liver ailments worldwide, with a prevalence rising in most developed countries [1]. NAFLD is a multifactorial disease that affects the liver and other peripheral organs and regulatory pathways and is characterized by excessive fat liver accumulations in the absence of alcohol consumption [2]. In Western countries, an incidence of NAFLD of around 20–30% of the adult population is estimated. The prevalence is lower in Eastern countries, but a rising tendency has been recently observed due to the changes in dietary habits, together with the sedentary lifestyle associated with Westernized societies [1]. These Western dietary habits are characterized by a predominant and excessive intake of saturated fats and caloric oversupply that, together with an impoverishment of lifestyle, are the key causes of developing NAFLD/NASH [3]. Moreover, lower consumption of vegetables, fruits, whole grains, ω3-fatty acids, and proteins facilitates NAFLD progression [3].

These chronic liver diseases are characterized by several pathologic features, including hepatic steatosis (HS), hepatocyte hypertrophy, and ballooning in NAFLD, together with fibrosis and inflammation in NASH [4,5]. Regarding the affected metabolic pathways associated with NAFLD progression, a mitochondrial dysfunctionality has been described. This alteration is caused by the imbalance between prooxidant and antioxidant mechanisms, which results in an increased production of reactive oxygen species (ROS), impairing the functionality of the electron transport chains and leading to an abnormal fatty acid oxidation [6,7]. Another key contributor to the pathogenesis of NAFLD is an increased visceral adipose tissue dysfunction, which is related to adipose hypertrophy, increased proinflammatory cytokine production and progressive fibrosis [8]. The adipose tissue dysfunction results in insulin resistance, limiting the amount of fat storage, and implicating a decreased de novo lipogenesis and increased lipolysis in the adipose tissue (due to inefficiency of insulin to block lipolysis) with consequent increased flux of fatty acids from adipose tissue to the liver [8,9]. This elevates free fatty acid (FFA) fluxes from adipose depots to the liver, which results in an increased hepatic de novo lipogenesis and an impaired inhibition of adipose tissue lipolysis [9]. These FFAs are accumulated in liver as triacyclglycerol (TG) and diacylglycerol (DAG). Increased production of DAGs promotes the synthesis of intermediates such as ceramides, which are associated with the development of high resistance in the hepatic insulin-signaling pathway through decreasing Akt activation. These metabolic alterations promote hepatic inflammation and the risk of progressive fibrosis. The synthesis of hepatic non-esterified fatty acids also induces endoplasmic reticulum stress, contributing to hepatic production of inflammatory cytokines as tumor necrosis factor-α (TNFα) [2].

Fatty liver is a primary stage of liver disease that is considered reversible and benign, whereas NASH is an advanced and more-dangerous phase that could progress to worse diagnoses such as cirrhosis and liver cancer [5,10]. Currently, there is no effective treatment for NAFLD and the main tools available to physicians are limited to behavioral changes focused on weight loss through the combination of exercise and a healthy diet [11,12]. Thus, novel medical or nutritional approaches are needed to fight against these diseases. 

Previous studies have shown that a multifactorial dietary intervention with bioactive cofactors that modulate the activity of the deregulated metabolic pathways can effectively revert hepatic steatosis in NAFLD patients [5]. This intervention intends to reduce liver fat content by increasing both mitochondrial fatty acid uptake and oxidation and glutathione (GSH) availability [5,13]. An integrated analysis performed by Mardinoglu et al., which combined personalized genome-scale metabolic models and in-depth multiomics profiling based on human data, indicated augmented requirements for nicotinamide adenine dinucleotide (NAD)^+^ and reduced GSH in patients with NAFLD, which could be related to this mitochondrial dysfunction [5,14,15]. Thus, the limited availability of serine and glycine in NAFLD patients reduced de novo GSH synthesis, protecting against ROS [7]. 

A clinical study in healthy subjects supplemented with a promising combination of metabolic cofactors to target fat liver accumulations and revert altered metabolic processes showed an improvement in blood inflammatory parameters, pointing out that this combination is a promising treatment for NAFLD [5,7,14]. These cofactors were present in a multi-ingredient mixture (MI) composed of L-carnitine (LC, an enhancer of fatty acid uptake across the mitochondrial membrane), n-acetyl cysteine (NAC) and serine (glutathione precursors protecting against ROS), and nicotinamide riboside (NR) as an NAD^+^ precursor [5,7,14]. Xia et al. described LC protective effects by accelerating FFAs transport into mitochondria in hepatocytes [16]. NR can increase NAD^+^ levels and accelerate oxidative metabolism and protection against obesity ameliorating metabolic disorders induced by impaired mitochondrial function [17]. NAC has a protective effect, reducing oxidative stress by increasing GSH availability, and can increase fatty acid re-uptake and fatty acid oxidation [13]. In other studies, serine was replaced by betaine, which is a methyl donor involved in methionine metabolism and may regulate cysteine levels, being a GSH precursor, because betaine can be converted to glycine and sarcosine [13,18]. These studies elucidated that supplementation with NAD^+^ and GSH precursors might be useful as a treatment of NAFLD, promoting fat oxidation in liver mitochondria, reducing liver fat content, increasing de novo GSH synthesis and improving liver function parameters [7].

To validate this hypothesis, a preclinical study in a well-established dietary mice model of NAFLD induced by high fat and high sugar consumption was performed [19]. This model had previously shown an aggravating effect of fructose on glucose and lipid metabolism, resulting in hepatic triglyceride accumulation, insulin resistance and a characteristic microvesicular, macrovesicular and steatosis effect similar to those described in humans [20,21]. In the present study, the impact of supplementation with this specific combination of metabolic cofactors was evaluated for the first time, focusing on the main features of NAFLD/NASH, liver steatosis, hepatic inflammation and hepatic insulin resistance.

## 2. Materials and Methods

### 2.1. Animal Model and Diets

Forty-eight C57BL/6J male mice (Envigo, Sant Feliu de Codines, Barcelona, Spain), 6 weeks old at the beginning of the experiment, were used (Figure S1). Animals were housed in groups (4 mice per cage) under controlled conditions of temperature (22 ± 2 °C) and humidity (55 ± 10%), and on a 12-h light/dark cycle with free access to food and water. Mice were left undisturbed to acclimate to the animal facility for one week. After the acclimatation period, animals were randomly divided into two experimental groups with different diets. Sixteen control mice were kept on a standard diet (D12328, Research Diets, New Brunswick, NJ, USA) and 32 animals (NAFLD group) were fed with HFHFr diet (HFHC: D12331, Research Diets) supplemented with 23.1 g/L fructose and 18.9 g/L sucrose in the drinking water. Mice were kept on these diets for a period of 20 weeks in ad libitum conditions. These specific doses were determined based on previous studies and a calculation of dose translation from human to animal dosage [22]. All experimental protocols were approved by the Animal Ethics Committee of the Technological Unit of Nutrition and Health of Eurecat (Reus, Spain) and the Generalitat de Catalunya approved all the procedures (10281). The experimental protocol followed the “Principles of Laboratory Care” guidelines and was carried out in accordance with the European Communities Council Directive (2010/63/EEC). 

For the last 4 weeks of the experiment (from the 16th to 20th week), NAFLD mice were randomly distributed into two groups: 16 mice were kept under the same fed conditions described before (HFHFr group), and 16 mice were exposed to multi-ingredient treatment (MI group). MI is a mix of the following compounds: 400 mg/kg of LC tartrate (Cambridge Commodities, Ely, UK), 400 mg/kg NAC (Cambridge Commodities), 800 mg/kg Betaine (Cambridge Commodities) and 400 mg/kg NR (ChromaDex, Los Angeles, CA, USA). LC was administrated through LC tartrate (LCT), containing 68.2% LC, providing 560 mg/kg to reach the dose of 400 mg LC/kg. Betaine, LCT, NAC and NR were diluted with drinking water with 23.1 g/L fructose and 18.9 g/L sucrose (vehicle). Fresh solutions were freshly prepared three times per week and prepared from stock powders and protected from light. Before being euthanized, 10 animals per group were randomly selected to perform an insulin challenge. They were intraperitoneally injected with 1 mU/g of insulin (*n* = 5 per group) or saline (*n* = 5 per group) and after 15 min, they were sacrificed. 

Serum was obtained by centrifugation and stored at −80 °C for further analysis. Serum alanine amino-transferase (ALT) and aspartate amino-transferase (AST) were quantified by enzymatic colorimetric assays (QCA, Barcelona, Spain). Fasting insulinemia and glycemia were measured with the Mouse Insulin ELISA Kit (Mercodia, Uppsala, Sweden) and the Glucose Liquid Kit (QCA, Barcelona, Spain), respectively. Livers were rapidly collected, weighed and divided into two sections—the lobus hepatis sinister medialis was kept in formalin, and the remaining tissue was frozen in liquid nitrogen and stored at −80 °C until further analysis.

### 2.2. Hepatic Fat Quantification

Hepatic lipids were extracted and quantified following a method previously described [23]. Briefly, total lipids were extracted from 80–100 mg liver sections by adding 1 mL of hexane/isopropanol (3:2, *v/v*) and degassing with gas nitrogen. Then, they were left overnight under orbital agitation, at room temperature, protected from light. After extracting with 0.3 mL of Na_2_SO_4_ (0.47 M), the organic layer was separated and dried with gas nitrogen. Total lipids were quantified gravimetrically before emulsifying, as described previously [24]. Triglycerides, total cholesterol, and phospholipids were measured using commercial enzymatic kits (QCA).

### 2.3. Histological Evaluation

Liver portions fixed in buffered formalin (4% formaldehyde, 4 gr/L NaH2PO4, 6.5 gr/L Na_2_HPO_4_; pH 6.8) were cut at a thickness of 3.5 μm and stained with hematoxylin & eosin (H&E) and trichrome stain. Liver images (magnification 40X) were taken with a microscope (ECLIPSE Ti; Nikon, Tokyo, Japan) coupled to a digital sight camera (DS-Ri1, Nikon) and analyzed using ImageJ NDPI software (National Institutes of Health, Bethesda, MD, USA; https://imagej.nih.gov/ij, version 1.52a). To avoid any bias in the analysis, the study had a double-blind design, preventing the reviewers from knowing any data from the mice during the histopathological analysis. A General NAFLD Scoring System was established to diagnose mice with NAFLD/NASH. The key features of NAFLD and NASH were categorized as follows: steatosis was assessed by analyzing macrovesicular (0–3) and microvesicular steatosis (0–3) separately, followed by hepatocellular hypertrophy (0–3), which evaluates abnormal cellular enlargement, and finally giving a total score of 9 points of steatosis state. Inflammation was scored by counting cell aggregates (inflammatory foci). The score 0 to 3 depends on the grade of the feature. It is categorized as 0 (<5%), 1 (5–33%), 2 (34–66%) and 3 (>66%), and this scoring is used in each feature of steatosis and then added to the total steatosis score [25]. Ballooning was not included in the scoring system, because only quantitative measures were considered for the rodent NAFLD score. It is important to highlight that hypertrophy is not a sign of cellular injury and slightly refers to an anomalous enlargement of the cells without recognizing the source of this enlargement [25]. Knowing that chronic inflammatory state might trigger fibrogenesis [26], liver fibrosis was analyzed by Masson’s trichrome-stained sections considering the collagen proportional area, as previously described [27,28].

### 2.4. RNA Extraction and Quantitative Polymerase Chain Reaction

Homogenates from 6 livers per groups were used for total RNA extractions using TriPure reagent (Roche Diagnostic, Sant Cugat del Vallès, Barcelona, Spain) according to the manufacturer’s instructions. RNA concentration and purity were determined using a nanophotometer (Implen GmbH, München, Germany). RNA was converted to cDNA using the High-Capacity RNA-to-cDNA Kit (Applied Biosystems, Wilmington, DE, USA). The cDNAs were diluted 1:10 before incubation with commercial LightCycler 480 Sybr green I master on a Lightcycler^®^ 480 II (Roche Diagnostics GmbH, Manheim, Germany). Table 1 shows a list of used primers that were previously described in other studies and verified with Primer-Blast software (National Center for Biotechnology Information, Bethesda, MD, USA). As previously described, 36b4 was used as a housekeeping gene [29,30,31].

### 2.5. Protein Extraction and Western Blot Analysis

Approximately 20 mg of liver was homogenized with Tyssuelyser LT (Qiagen, Hilden, Germany) for 50 s in 300 µL lysis buffer (8 mmol/L NaH_2_PO_4_, 42 mmol/L Na_2_HPO_4_, 1% SDS, 0.1 mol/L NaCl, 0.1% NP40, 1 mmol/L NaF, 10 mmol/L sodium orthovanadate, 2 mmol/L PMSF, and 1% protease inhibitor cocktail 1 (Millipore Sigma, Darmstadt, Germany)). The protein extracts were quantified by the standardized BCA method (Bio-Rad Protein Assay; BioRad, Hercules, CA, USA). Protein extracts (20–25 µg) were electrophoretically separated on 10% SDS-PAGE and electroblotted to nitrocellulose membranes (Li-cor biosciences, NE, USA) [32]. Efficient protein transfer was monitored by Ponceau-S stain. Next, membranes were blocked (5% BSA) at room temperature and probed with specific primary antibodies (diluted 1:1000) overnight at 4º C in 1% BSA: total Akt (4685) (CST, Danvers, MA, USA), phospho-Akt (Ser473) (4060) (CST) and β-Actin (Santa Cruz Biotechnology, Inc.; Dallas, TX, USA). Thereafter, infrared fluorescent secondary antibodies anti-rabbit 680, anti-rabbit 800 and anti-mouse 680 (LI-COR Biosciences, Lincoln, NE, USA; 926-32211, 926-68071 and 926-68070, respectively) were used for detection and quantified using ImageJ [33]. 

**Table 1:** Sequences of the used RT-PCR oligonucleotides.

Primers	Forward	Reverse	Reference
Ppara	5′-CCCTGTTTGTGGCTGCTATAATTT-3′	5′-GGGAAGAGGAAGGTGTCATCTG-3′	[34]
Fasn	5′-GCTGCGGAAACTTCAGGAAAT-3′	5′-AGAGACGTGTCACTCCTGGACTT-3′	[35]
Col1a1	5′-TAGGCCATTGTGTATGCAGC-3′	5′-ACATGTTCAGCTTTGTGGACC-3′	[36]
Tnfa	5′-AGGGTCTGGGCCATAGAACT-3′	5′-CCACCACGCTCTTCTGTCTAC-3′	[36]
Il6	5′-AGTTGCCTTCTTGGGACTGA-3′	5′-TCCACGATTTCCCAGAGAAC-3′	[37]
Il1a	5′-CCAGAAGAAAATGAGGTCGG-3′	5′-AGCGCTCAAGGAGAAGACC-3′	[38]
F4/80	5′-CATAAGCTGGGCAAGTGGTA-3′	5′-GGATGTACAGATGGGGGATG-3′	[39]
Scd1	5′-AGATCTCCAGTTCTTACACGACCAC-3′	5′-GACGGATGTCTTCTTCCAGGTG-3′	[40]
Acc1	5′-GATGAACCATCTCCGTTGGC-3′	5′-CCCAATTATGAATCGGGAGTGC-3′	[40]
Cd36	5′-GAACCACTGCTTTCAAAAACTGG-3′	5′-TGCTGTTCTTTGCCACGTCA-3′	[40]
Fabp4	5′-TGAAAGAAGTGGGAGTGGGC-3′	5′-CGAATTCCACGCCCAGTTTG-3′	[41]
Cpt1a	5′-CTCAGTGGGAGCGACTCTTCA-3′	5′-GGCCTCTGTGGTACACGACAA-3′	[42]
Cbs	5′-GCAGCGCTGTGTGGTCATC-3′	5′-CATCCATTTGTCACTCAGGAACTT-3′	[43]
Fgf21	5′-CCTCTAGGTTTCTTTGCCAACAG-3′	5′-AAGCTGCAGGCCTCAGGAT-3′	[44]
Ucp2	5′-GGTCGGAGATACCAGAGCAC-3′	5′-ATGAGGTTGGCTTTCAGGAG-3′	This study
Glut2	5′-ACCCTGTTCCTAACCGGG-3′	5′-TGAACCAAGGGATTGGACC-3′	[45]
G6pd	5′-GTGGGATCCTGAGGGAAGAGT-3′	5′-GATGGTGGGATAGATCTTCTTCTTG-3′	[34]
Srebp1c	5′-TGACCCGGCTATTCCGTGA-3′	5′-CTGGGCTGAGCAATACAGTTC-3′	[46]
Ucp1	5′-ACTGCCACACCTCCAGTCATT-3′	5′-CTTTGCCTCACTCAGGATTGG-3′	[47]
36bB4	5′-AGTCCCTGCCCTTTGTACACA-3′	5′-CGATCCGAGGGCCTCACTA-3′	[48]

### 2.6. Statistical Analysis

Statistical analyses were performed using GraphPad Prism 9 software (Graph-Pad Soft-ware, La Jolla, CA, USA). Data are presented as mean ± SEM. Data distribution was analyzed by the Shapiro–Wilk normality test. Differences between two groups were deter-mined using an unpaired *t*-test (two-tailed, 95% confidence interval). One-way analysis of variance (ANOVA) was conducted to examine differences between three groups. A *p*-value below 0.05 was considered statistically significant.

## 3. Results

### 3.1. MI Supplementation Reduced Liver Injury and Macroscopic Liver Features of NAFLD

**Figure 1:**
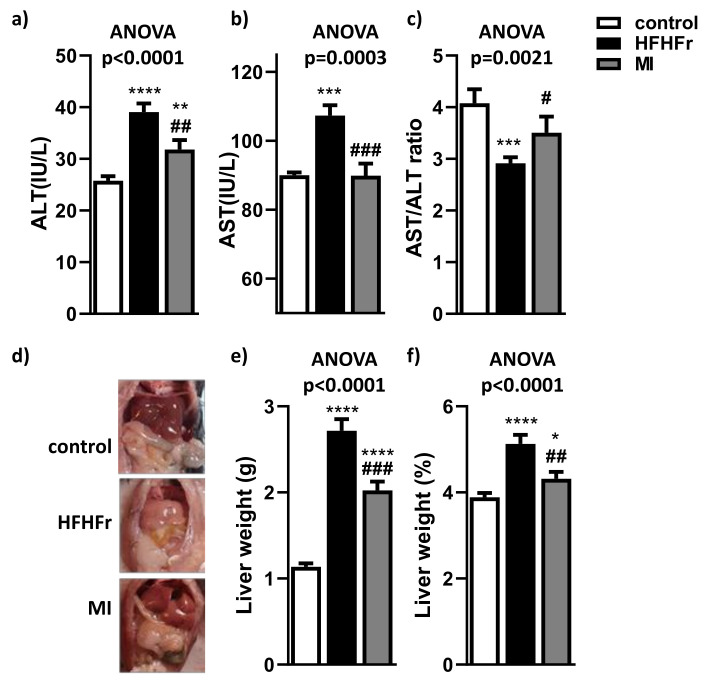
Effects of treatments on (**a**) serum ALT (alanine amino-transferase); (**b**) serum AST (aspartate amino-transferase); (**c**) serum AST/ALT ratio; (**d**) representative macroscopic appearance of livers; (**e**) liver weight; and (**f**) liver weight/body weight (%). Data are mean ± SEM; *n* = 16 animals/group. * *p* < 0.05, ** *p* < 0.01, *** *p* < 0.001, **** *p* < 0.0001 vs. control mice; ^#^ *p* < 0.05, ^##^ *p* < 0.01, ^###^ *p* < 0.001 vs. HFHFr mice.

After 4 weeks of treatment, MI supplementation (treatment composed of betaine, NR, NAC and L-carnitine) promoted a reduction in body weight (Figure S2a) and a tendency to reduce adiposity (Figure S2b). These reductions were not a consequence of food intake decrease, because no differences were observed between MI and HFHFr groups (Figure S2c,d). Liver injury was evaluated by measuring ALT and AST serum levels. Both transaminases were clearly increased in serum of HFHFr mice (Figure 1a,b). In contrast, MI mice presented a decreased level of both transaminases (Figure 1a,b), almost to control mice levels, lowering these liver injury markers at similar levels than non-NAFLD animals (HFHFr group). The AST/ALT ratio was significantly decreased in the HFHFr mice group compared to control mice. However, the AST/ALT ratio was significantly recovered in MI supplemented mice close to control group results (Figure 1c). In addition, macroscopic assessment showed fatty liver appearance in HFHFr animals compared to the control group, whereas MI intervention partially recovered a healthy liver appearance in comparison with HFHFr animals (Figure 1d). In fact, MI supplementation significantly attenuated the liver weight increase observed in HFHFr group (Figure 1e). This phenomenon was also observed in liver weight relative to body weight (Figure 1f). 

### 3.2. MI Supplementation Decreased Hepatic Lipid Content and Liver Steatosis

**Figure 2:**
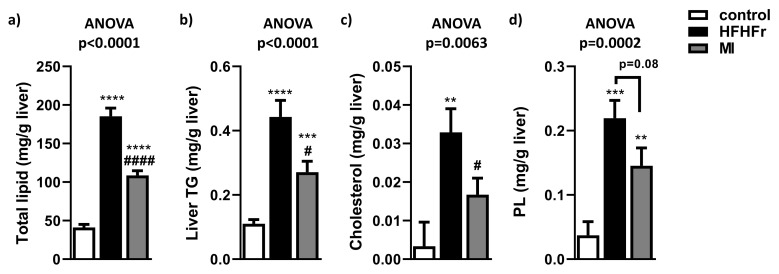
Effects of treatments on (**a**) total liver lipid content; (**b**) total hepatic triglyceride (TG) content; (**c**) total hepatic cholesterol content; and (**d**) total hepatic phospholipid (PL) content. Data are mean ± SEM; *n* = 16 animals/group. ** *p* < 0.01, *** *p* < 0.001, **** *p* < 0.0001 vs. control mice; ^#^ *p* < 0.05; ^####^ *p* < 0.0001 vs. HFHFr mice.

To determine if MI intervention reduced liver lipid accumulation, hepatic lipid content was evaluated. As expected, hepatic lipid content was increased in the HFHFr group compared to the control group (Figure 2a), and MI treatment significantly reduced this effect observed in HFHFr mice. A similar pattern was observed in hepatic triglyceride content (Figure 2b), in hepatic cholesterol content (Figure 2c), and in phospholipid content (Figure 2d), observing that the HFHFr diet significantly increased these lipid species compared to the control group counterparts. In contrast, MI supplementation significantly reduced hepatic triglyceride and cholesterol content and showed a tendency to reduce phospholipid levels.

**Figure 3:**
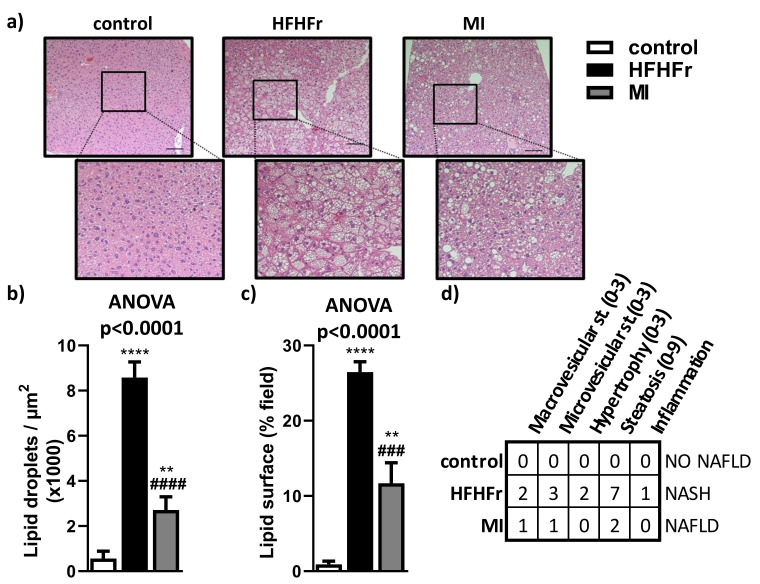
Liver histopathology and image analysis were determined by (**a**) H&E (hematoxylin-eosin staining) and an amplification of the selected area showing a magnified area in the lower panel; (**b**) lipid droplets count; (**c**) lipid droplet surface field; and (**d**) NAFLD/NASH scoring table. st., steatosis. Bar = 100 µm. Data are mean ± SEM; *n* = 6 animals/group. ** *p* < 0.01, **** *p* < 0.0001 vs. control mice; ^###^ *p* < 0.001; ^####^ *p* < 0.0001 vs. HFHFr mice.

Consistent with biochemical analysis, liver histology analysis showed that the HFHFr group developed pronounced liver steatosis by the presence of micro- and macrovesicular steatosis with nuclear displacement due to hypertrophy (Figure 3a–c). Interestingly, treatment with MI ameliorated liver steatosis, as microvesicular steatosis was almost absent, macrovesicular steatosis was markedly reduced, there was a pronounced reduction in the amount of lipid droplets (Figure 3a–c) and hypertrophy and disturbance of nucleus were diminished. These results, corroborated by the general NAFLD Scoring System, demonstrate a noticeable amelioration of different parameters analyzed in animals treated with MI supplementation compared with the HFHFr group (Figure 3d).

### 3.3. Beneficial Effects of MI Supplementation Did Not Involve Lipogenesis, Lipid Transport or Fatty Acid Oxidation Pathways

**Figure 4:**
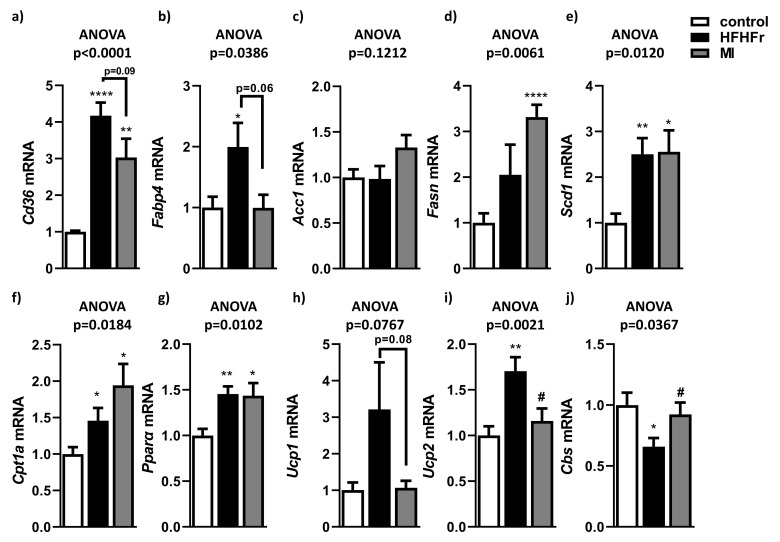
Hepatic mRNA expression of genes related to lipid transport, (**a**) Cd36 and (**b**) Fabp4, de novo hepatic lipogenesis (**c**) Acc1, (**d**) Fasn and (**e**) Scd1 and fatty acid oxidation (**f**) Cpt1a and (**g**) Pparα; antioxidative defense (**h**) Ucp1, (**i**) Ucp2 and (**j**) Cbs. Data are mean ± SEM. *n* = 6 animals/group. * *p* < 0.05, ** *p* < 0.01, **** *p* < 0.0001 vs. control mice; ^#^ *p* < 0.05 vs. HFHFr mice.

To discern which metabolic pathways could be involved in NAFLD improvement after MI supplementation, analysis of gene expression related to hepatic lipogenesis, lipid transport and fatty acid oxidation were carried out. The HFHFr group showed a significant increase in lipid transport-related genes Cd36 (Figure 4a) and Fabp4 (Figure 4b). Interestingly, a tendency to decrease expression of Cd36 and Fapb4 was observed in MI-supplemented mice compared to the HFHFr group (Figure 4a,b). No significant changes were observed in mRNA levels of the genes involved in de novo hepatic lipogenesis, Acc1 (Figure 4c) and Fasn (Figure 4d). Although expression of Scd1 was induced in the HFHFr and MI groups compared with the control group, there was no difference between both groups (Figure 4e). Regarding fatty acid oxidation, increased expression of Cpt1a (Figure 4f) and Pparα (Figure 4g) was observed in the HFHFr group compared with the control group, but no significant differences were observed in the supplemented MI group compared with the HFHFr group. In contrast, hepatic expression levels of the main uncoupling proteins (Ucp1 and Ucp2) were increased in the HFHFr group, in comparison with the control group (Figure 4h,i). However, after MI supplementation, hepatic expression of Ucp1 and Ucp2 reverted to similar levels as in control mice. In addition, hepatic Cbs expression levels (a key enzyme in GSH levels to defense against oxidative stress) were downregulated in the HFHFr animals (Figure 4j). In contrast, animals treated with the MI supplementation reversed this downregulation, with hepatic Cbs expression levels similar to control animals (Figure 4j).

### 3.4. MI Supplementation Reduced Hepatic Inflammation Associated to NAFLD

**Figure 5:**
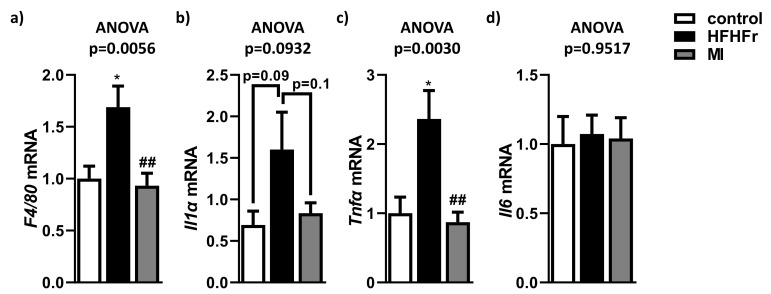
Effects of treatments on (**a**) expression of F4/80, (**b**) Il1α, (**c**) Tnfα, (**d**) Il6. Data are mean ± SEM. *n* = 6 animals/group. * *p* < 0.05 vs. control mice; ^##^ *p* < 0.01 vs. HFHFr mice.

To evaluate whether supplementation with MI modulated the levels of proinflammatory markers, the expression levels of some representative inflammatory genes were assessed. As expected, F4/80 mRNA levels, a marker of macrophage infiltration, were significantly upregulated in the HFHFr group compared to control mice (Figure 5a). In contrast, MI treatment significantly corrected this upregulation of F4/80 expression. Similar results were observed in the expression of Il1α (Figure 5b) and Tnfα (Figure 5c), which was upregulated in HFHFr animals but supplementation with MI reversed it to levels analogous to the control group. However, in the case of Il1α, it was just a statistical tendency. Moreover, the expression of Il6 did not show any differences between groups (Figure 5d).

### 3.5. MI Supplementation Reduced Fibrosis Markers Associated to NAFLD

**Figure 6:**
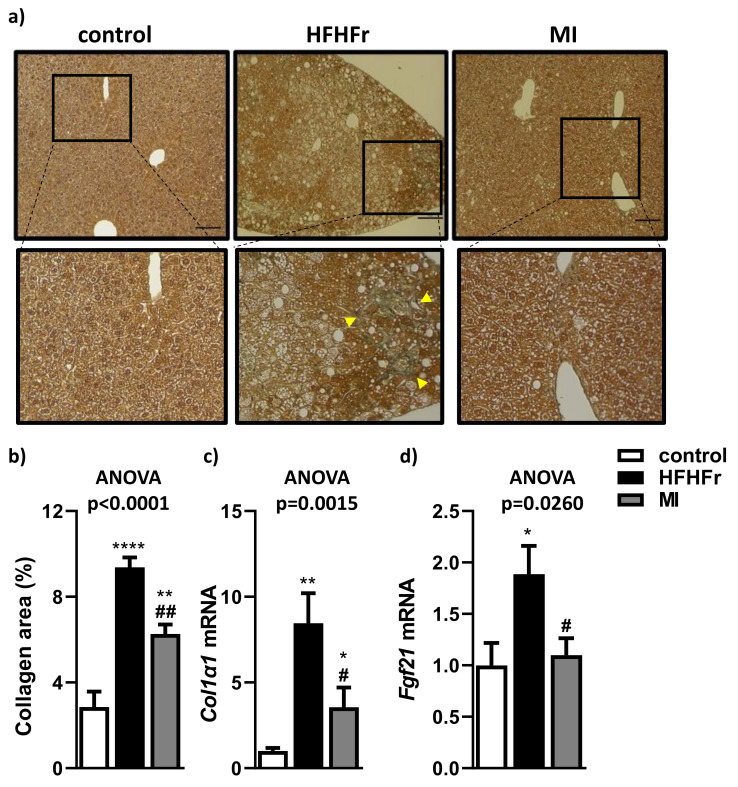
Effects of treatments on hepatic fibrosis. (**a**) Representative images of Masson’s trichrome staining showing a magnified area in the lower panel; (**b**) quantification of collagen fiber area; (**c**) hepatic mRNA expression of Col1α1 and (**d**) Fgf21. Bar = 100 µm. Data are mean ± SEM. *n* = 6 animals/group. * *p* < 0.05, ** *p* < 0.01, **** *p* < 0.0001 vs. control mice; ^#^ *p* < 0.05, ^##^ *p* < 0.01 vs. HFHFr mice. Yellow arrowheads show the fibrotic structures.

Liver fibrosis was analyzed by Masson’s trichome staining and quantified through the number of collagen fibers stained afterwards. These liver sections showed an increase in the fibrotic state in the HFHFr group compared to control group due to an increase in stained collagen fibers (Figure 6a,b), although the MI-supplemented group showed a better % of area occupied by collagen in comparison with the HFHFr group. Indeed, hepatic expression of the pro-fibrotic gene Col1α1 was significantly increased in the HFHFr group in comparison with the control group (Figure 6c). In contrast, supplementation with MI significantly reduced Col1α1 and Fgf21 hepatic levels compared to the HFHFr group [49,50] (Figure 6c,d).

### 3.6. MI Supplementation Reduced Hepatic Insulin Resistance Associated to NAFLD

**Figure 7:**
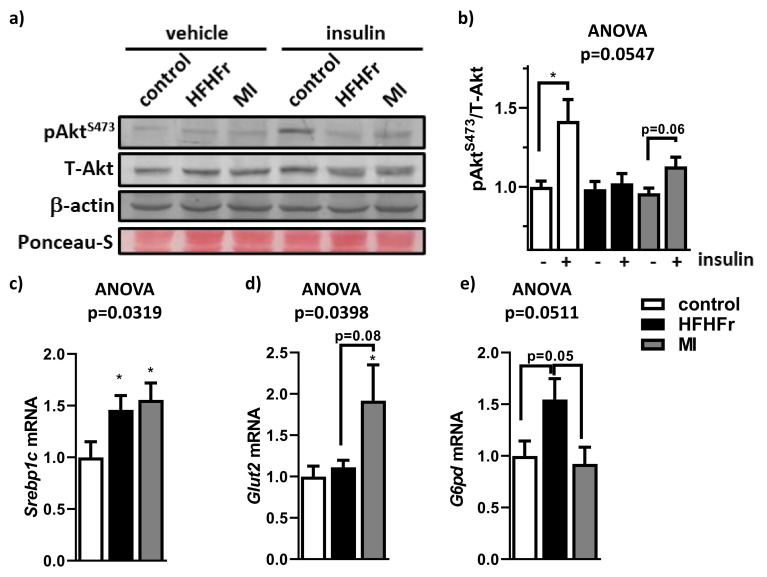
Effects of treatments on liver insulin resistance. (**a**) A representative Western blot analysis with Akt activation (pAkt^S473^), total Akt protein levels (T-Akt), housekeeping β-actin levels and protein loading with Ponceau-S membrane staining; (**b**) densitometry analysis of phosphorylated and total Akt ratio. Hepatic mRNA expression of: (**c**) Srebp1, (**d**) Glut2 and (**e**) G6pd. Data are mean ± SEM. *n* = 5–6 animals/group. * *p* < 0.05 vs. control mice.

Considering that impaired hepatic insulin signaling plays an important role in NAFLD development, in order to evaluate insulin resistance, animals were challenged with an intraperitoneal insulin bolus determining Akt activation (phosphorylation on Serine 473) or saline. As expected, control group showed a significant Akt phosphorylation (pAkt^S473^) in insulin challenged mice compared to vehicle injected mice (Figure 7a,b). This fact was not observed in the HFHFr group, indicating hepatic insulin resistance in these animals. In contrast, the MI-supplemented group showed a tendency to improve insulin signaling (Figure 7a,b). In addition, systemic fasting glycaemia and fasting insulinemia were improved after the MI supplementation (Figure S3a,b). To validate the amelioration of hepatic insulin signalling, we evaluated the liver expression of a key transcription factor involved in the insulin response, Srebpc1 (Figure 7c); the main hepatic glucose transporter, Glut2 (Figure 7d); and an important regulator of the lipid and carbohydrate metabolism, G6pd (Figure 7e). As expected, Srebp1c expression was increased in the HFHFr group compared with the control group (Figure 7c). Similar results were observed in the MI-supplemented animals. In contrast, Glut2 expression was increased in the MI-supplemented group (Figure 7d). Finally, G6pd expression tended to be higher in the HFHFr group compared with the control animals, whereas this effect was almost completely abolished in the MI-supplemented group (Figure 7e).

## 4. Discussion

NAFLD is a multifactorial disease, which is related to obesity, dyslipidemia and insulin resistance, all risk factors that can favor the development of this disease [51]. Novel medical or nutritional approaches to modulate the different alterations associated with NAFLD are needed. In this study, the effect of a multi-ingredient supplementation was evaluated as a treatment for this pathology. MI consisted of a mix of NAC, NR, LC and betaine. In this study, betaine was used instead of serine because of its efficiency to promote mitochondrial fatty acid absorption and GSH biosynthesis through the folate cycle, which can be converted to sarcosine and glycine, leading to a decrease in liver fat content [5,7,13]. In this line, a study with glycine n-methyltransferase-deficient mice (glycine n-methyltransferase participates in the catabolism of betaine to glycine) showed that these deficient mice developed hyperlipidemia and steatohepatitis [52]. Furthermore, in a clinical study NAC, LC and betaine plasma levels were negatively correlated with hepatic steatosis [53], and the administration of GSH and NAD^+^ precursors significantly decreased hepatic steatosis in a preventive treatment study in mice [53]. Supplementation with MI is supported by previous studies describing an increase in fat oxidative metabolism in the liver after the administration of this combination in patients suffering from NAFLD [14]. 

In the present study, after NAFLD was established [54], the effects of MI supplementation for 4 weeks were evaluated. The MI-supplemented group showed significant reductions in systemic AST and ALT levels compared to their HFHFr counterparts, improving liver injury associated to NAFLD. These results correlate with previous clinical studies with NAC and LC, which showed a reduction in circulatory levels of transaminases [55,56]. However, there are some inconsistencies in the literature about the alterations in the AST/ALT ratio associated with NAFLD. In the present study, HFHFr mice exhibited a decreased AST/ALT ratio compared to the control group, which was consistent with other studies in obese and NAFLD mouse models fed with a high-fat, high-fructose diet [57,58,59,60,61]. However, there are some discrepancies between our results and other studies with an increased AST/ALT ratio due to NAFLD and different circulating transaminases levels that must be considered [62]. Therefore, more efforts are needed to discern AST/ALT relationships in the context of NAFLD. Although liver weight was increased in this model of NAFLD [19], 4 weeks with MI supplementation decreased liver weight. As expected, the HFHFr group showed a significant increase in the severity of HS compared to control mice, with higher hypertrophy, presence of inflammatory foci and disturbance of nucleus [54]. Interestingly, these findings were accompanied by a significant improvement in all markers of hepatic steatosis evaluated in the MI-supplemented group. These findings are in agreement with recent investigations where HS was lowered by either serine treatment in a clinical study, as well as by a combination of NR, NAC and serine in a preclinical study [14]. 

Furthermore, a glycine-based treatment in animal models with liver damage has been observed to protect against oxidative stress mediated by free radicals, which could reduce cellular damage and the presence of inflammatory and fibrotic foci [63,64]. Interestingly, NAD+, which was found increased by the presence of its precursor NR, protected against steatosis in mice [17,65], and triggered inflammation [66]. These NAD+ and GSH-based studies suggested that MI supplementation would be a promising strategy to reduce fatty liver. In NAFLD, hepatic overload of FFAs and fatty acid oxidation impairment are central to its pathogenesis. When FFA are delivered to the liver, or hepatic de novo lipogenesis exceeds triglycerides export or oxidation, NAFLD might progress [67]. In agreement with other preclinical studies, the HFHFr group presented a significant increase in total hepatic lipids, triglycerides and cholesterol compared to the control group [23,68]. On the contrary, MI-supplemented mice showed a significant reduction in these lipid parameters. These results agree with a preclinical study, in which obese mice were supplemented with a similar nutritional cocktail composed of NR, NAC and serine, decreasing hepatic lipid parameters [14]. The first two components would stimulate FFA transfer from cytosol to the mitochondria and raise NAD^+^ levels, thereby increasing mitochondrial FFAs oxidation. The last two components would help increase GSH levels, protecting against ROS [19]. 

Fatty acid transport into the liver occurs via fatty acid transporters such as fatty acid translocases (Cd36) and fatty acid binding proteins (Fabp4) [69]. The expression of these genes was positively related to liver fat content, developing hepatosteatosis and hepatic insulin resistance [70]. Interestingly, the tendency to decrease hepatic expression of Cd36 and Fabp4 found in response to MI supplementation suggests that this multi-ingredient treatment would tackle NAFLD by reducing FFA uptake into the liver. Different previous studies contribute to reinforce this hypothesis. Thereby, a preclinical study showed that FABP expression tended to increase in obesogenic studies, but a methyl donor supplementation may revert this rise [71], such as betaine, which is a methyl donor in the MI treatment. Cd36 was shown as a possible target of NAC, since NAC treatment in male rats with NASH displayed a reduction in Cd36 expression [72]. NR supplementation also showed a lowering effect on Cd36 mRNA expression in an experimental NAFLD mice model related to amelioration of the disease [65]. Therefore, the tendency to reduce expression of fatty acid transport-related genes is correlated with a reduction in fatty liver.

Regarding hepatic lipogenesis (Fasn, Acc1 and Scd1) and fatty acid oxidation (Cpt1a and Pparα), there were no significant differences between MI-supplemented mice and their HFHFr mice counterparts. However, previous studies showed opposite results for these determinations. For example, *Fasn* mRNA expression, a key enzyme involved in liver de novo lipogenesis, did not show significant differences in a previous mice study treated with an NR, NAC and L-serine cocktail [14]. In contrast, administration of NR in a fatty liver mice model showed a significant reduction in Fasn, Acc1 and Scd1 expression [65]. Pparα gene expression (a regulator of mitochondrial biogenesis and contributor to better NR function by enhancing β-oxidation and mitochondrial biogenesis) was increased in the MI group, but also in the HFHFr group compared to the control group. According to another preclinical study, in which NAFLD mice were treated with a combination of cofactors (including NR), no changes in Pparα expression were observed in the NAFLD group and treated group [65]. Therefore, the main effects observed by supplementation with MI do not appear to be related neither to liver lipogenesis nor to fatty acid oxidation.

Liver inflammation is a key feature of NAFLD, especially in the NASH stage. There are several altered processes that activate the inflammatory pathways triggering the progression to steatohepatitis [73], which include intrahepatic accumulation of several lipid species: increased lipid peroxidation, Kupffer cells activation by ROS accumulation and hepatic infiltration of macrophages [74]. Moreover, macrophage and Kupffer cell infiltration contribute to the progression of NAFLD and insulin resistance secreting pro-inflammatory cytokines such as TNF-α, IL-6 and IL-1β. Therefore, macrophage infiltration and inflammatory cytokines release increase hepatic lipid accumulation, which leads to dysregulation of lipid metabolism and exacerbates insulin by degradation of insulin receptor substrate 1 (IRS1) [75,76]. Hence, F4/80 expression, which is a macrophage marker, is increased in HFHFr group compared to control group. In this sense, supplementation with MI significantly attenuated the expression of the macrophage infiltration marker F4/80 observed in HFHFr group. These results are consistent with the previously reported reduced macrophage infiltration as a response to a glycine-based treatment, which was assessed by histological studies in livers of NASH mice [64]. Similar results, regarding decreased F4/80 presence in liver sections of NAFLD mice, were observed in a study using NR-based treatment [77]. In concordance with lower macrophage infiltration, supplementation with MI promotes a downregulation of some pro-inflammatory cytokines. Thus, Tnfα expression was also attenuated in the MI-supplemented group, suggesting that this cocktail was helpful in preventing inflammation of NAFLD in mice. This effect was also observed in other clinical and preclinical NASH studies, presenting a significant regression of Tnfα mRNA expression after L-carnitine supplementation [56], after glycine treatment [64] or in NR-treated mouse livers [65,77,78], supporting the hypothesis that NR treatment improves hepatic inflammation by modulating pyrin domain containing 3 (NLRP3) inflammasome. In addition, Il1α was also described to be up-regulated in patients with NAFLD [79] and, in the present study, Il1α tended to be elevated in HFHFr mice, but in the MI-supplemented group, this cytokine tended to be down-regulated, suggesting an anti-inflammatory effect. Unexpectedly, no significant effects were observed in Il6 mRNA expression in mice between the three groups. Indeed, data on the hepatic expression of *Il6* are contradictory. There are cases where treatments decrease this cytokine’s levels: with NR [65,78] or with betaine [80] and others, treated with LC and NR, no significant impact on the hepatic expression of Il6 was observed [81]. 

Several reports have strongly suggested that NAFLD could activate fibrotic pathways in advanced stages such as NASH [1,26]. In the present study, the anti-fibrotic effect of MI supplementation was evaluated in the context of NAFLD. Results showed that Col1α1 expression (the most abundant type of collagen found in scar tissue [82]), which was importantly increased in HFHFr group, was significantly reduced in MI-supplemented group. These findings were corroborated by the histological Masson’s trichome staining analysis, showing a significant reduction in collagen fibers due to MI treatment. These antifibrotic effects are in consonance with other NAFLD/NASH studies where NR treatment prevented the abnormal expression of hepatic Col1α1 and reduced Masson’s trichome staining in liver sections [65,83]. In addition, a recent study showed that glycine-based supplementation attenuated hepatic Col1α1 expression in NASH mice [64]. Taken together, supplementation with MI could be efficient as a treatment even in advanced stages of NAFLD/NASH. Betaine increase folate and methionine metabolism, which are closely related to an increased GSH production [7]. To check betaine’s effect in the MI supplementation, the hepatic expression of Cbs expression, which is translated as a key enzyme in GSH production [84], was analyzed in each group. HFHFr mice showed a significant decrease in Cbs expression compared to the control group. A deficient expression of Cbs was related to hepatic steatosis, inflammation, and fibrosis [84]. In contrast, the MI group significantly recovered Cbs expression in comparison with the HFHFr group, reaching similar levels to control animals. These results could be related to an increased antioxidant response due to betaine supplementation, concordant with another preclinical study [85]. Fgf21 and Ucp2 mRNA expression were assessed in order to evaluate the protective role of these molecules against inflammation and fibrosis. Fgf21 was overexpressed as a protective response against inflammation by obesogenic diets [49,86]. Moreover, ER stress, oxidative stress and lipotoxicity stimulate Fgf21 expression in NAFLD [87,88]. Concordant with previous studies, Fgf21 mRNA expression was upregulated in the HFHFr group, elucidating the protective response of hepatocytes against hepatic inflammation and fibrosis, which may end up in hepatocellular carcinoma [49]. The increased Fgf21 expression could be linked to Pparα upregulation and may suggest a need for increased hepatic FGF signaling as a protective response to NAFLD [86,89]. In the MI group, Fgf21 upregulation was mitigated compared to the HFHFr group and it could be related to a reduction in oxidative stress, increasing glutathione biosynthesis and antioxidant responses by betaine supplementation, which reduced the ratio of oxidized/reduced glutathione [90]. Therefore, this decrease in Fgf21 levels could be related with the amelioration of NAFLD progression by MI supplementation. In the case of Ucp2, it plays a role in proton leak through the mitochondrial membranes and prevents ROS production. Hepatic accumulation of FFAs facilitates the development and progression of NAFLD by impairing mitochondrial function, exceeding NADH in the mitochondrial matrix that needs to be re-oxidized [91]. The wastage of this mitochondrial membrane potential is a hallmark of mitochondrial dysfunction in NAFLD [92]. Hepatocytes up-regulate Ucp2 as an adaptation to obesity, but cells become vulnerable to ATP depletion and these cells are vulnerable to necrosis [93]. The HFHFr group showed a significant increase in this gene expression compared to the control group. These results are concordant with another preclinical study [94]. On the other hand, MI-supplemented mice showed a significant decrease in Ucp2 expression compared to the HFHFr group, in accordance with a preclinical study where NR caused a significant decrease in Ucp2 mRNA levels in obese mice, which was related to improved oxidative metabolism and protection against high-fat diet-induced metabolic abnormalities [17]. An assessment of plasma acylcarnitine would have been of interest to determine the effects related to fatty acid processing and β-oxidation due to LC and NR from MI treatment. Salic et al. [81] demonstrated, in an experimental NAFLD mice model, elevated acylcarnitine plasma levels and loss of fat were correlated, mimicking some processes that are also activated during exercise, leading to lipid oxidation. Indeed, the researchers assessed expression levels of some representative inflammatory genes and evaluated hepatic steatosis and fibrosis, which are related to oxidative stress and the accumulation of ROS, as Ali et al. explained [95]. However, considering that Laurent et al. [96] demonstrated how NAC treatment reduces oxidative stress and increases intracellular GSH levels in an experimental NAFLD mice model, and that Khodayar et al. [18] showed how betaine reduces mitochondrial ROS, regenerates mitochondrial GSH levels and increases antioxidant defense capacity, our results point the antioxidant activity of NAC and betaine. 

Insulin resistance is critical for the progression of NAFLD. The first hit is when increased dietary intake leads the body to produce excess fatty acids induced by lipogenesis and FFA synthesis, and they circulate in peripheral tissues, including the liver. The second hit occurs when hepatic FFAs accumulation is combined with oxidative stress, lipid peroxidation and mitochondrial dysfunction, contributing to insulin resistance [75,76]. Previous studies in humans and animals demonstrated that LC administration improved insulin sensitivity related to reduction in steatosis [56,73]. Insulin regulates glucose and lipid metabolism, where the PI3K/Akt pathway is their main effector. Thus, the PI3K/Akt pathway malfunction leads to insulin resistance, which is indeed linked to obesity and type 2 diabetes [97]. In the present study, a pronounced hepatic insulin resistance was observed in the HFHFr group. Nevertheless, the MI-supplemented group showed a tendency to improve insulin signaling. These results are in accordance with an in vitro study using insulin-resistant HepG2 cells, where betaine addition restored Akt activation [98]. This suggests that the mix of bioactive cofactors used in this study was helpful to recover hepatic insulin sensitivity and, therefore, the progression to NAFLD. Insulin resistance could be caused by inhibition of insulin receptor substrate-1 (IRS-1), and it could impair glucose transporter 2 (GLUT2) translocation in hepatocytes [99]. No significant differences were found in Glut2 mRNA expression between the HFHFr group and the control group, despite steatosis in HFHFr mice. However, circulating glucose and insulin are increased in HFHFr mice compared to control group, increasing insulin resistance. The reduced phosphorylation of Akt resulted in reduced activation of insulin signaling, which is related to impaired Glut2 expression. In contrast, the MI group showed tendency to increase Glut2 expression compared to HFHFr mice, accompanied by a reduction in circulating glucose and insulin levels and correlated with the tendency to improve Akt phosphorylation. These effects reduced the circulating glucose and insulin levels and improved insulin signaling [81], which could mean that MI supplementation induces Glut2 expression as an insulin sensitizing action. These results suggest that the mix of bioactive cofactors used in this study was helpful to recover hepatic insulin sensitivity and attenuate the progression to NAFLD. Srebp1c expression (which is closely related to Akt activation, lipogenesis in hepatocytes and is regulated by insulin signaling) was analyzed in each group [100,101,102]. SREBP1c plays an intermediary role in lipid homeostasis by orchestrating the gene transcription of enzymes involved in lipogenesis and lipolysis [100]. The HFHFr group showed an increased *Srebp1c* expression compared to the control group, agreeing with previous studies with obese animal models [103]. Srebp1c overexpression in mice with fatty livers was related to significant increases in lipid biosynthesis gene expression after *Srebp1c* activation, such as G6pd [104,105,106]. G6pd is a lipogenic gene linked to *Srebp1c*, which acts as a key transcriptional factor [107]. A decreased *Srebp1c* expression in the MI group compared to the HFHFr group was expected in a study with mice models with liver injuries treated with NR, and another study with an experimental NAFLD rat model treated with betaine [80,108]. However, the MI group did not show a significant difference in *Srebp1c* expression compared to the HFHFr group, probably because a longer treatment time was necessary to see the effects on the regulation of this gene. Despite this, a significant difference in G6pd expression being downregulated in MI supplementation was observed, which could indicate an amelioration in lipid homeostasis by decreasing lipogenesis. Indeed, increased expression of G6pd promotes hepatic steatosis and insulin resistance [109], but in the MI group, G6dp expression is decreased, which could be ameliorating this effect. G6pd expression tended to be higher in the HFHFr group compared to the control group (Figure 7). Contrarily, the expression of G6pd in MI-supplemented animals tended to be lower than the HFHFr group, achieving similar levels to the control group, which could point to an amelioration in lipid homeostasis by decreasing lipogenesis.

There are some limitations of this observational study. First, the lack of use of single-housing and paired-feeding techniques to control food intake individually in mice. However, social housing is essential for rodents, so housing them in individual cages is discouraged [110]. Second, conclusions derived from the present study are sustained in young male mice. Although this situation occurs commonly in other studies [14,81,111], it is necessary to validate the effect of supplementation with MI in other models, both in older mice and in females. Third, the present study lacks an MI-treated group without NAFLD, but the study design was similar to other studies [14,81,112] and no deleterious effect was expected for this treatment. Fourth, more studies with NASH models are needed to obtain more accurate information on the impact of MI supplementation on liver fibrosis.

## 5. Conclusions

To summarize, this study is the first to use a combination of cofactors composed of NAC, NR, betaine, and LC as a possible dietary strategy to treat NAFLD development in an animal model due to its implication in different metabolic pathways that are pathologically affected in this disease. Data from the present study suggest that 4-week supplementation with this specific combination of bioactive cofactors, at intended human clinical doses, ameliorates NAFLD features in mice. These improvements involved a reduction in liver weight, hepatic steatosis, inflammation, fibrosis and partially improved insulin sensitivity. Hepatic lipid metabolism is modulated through a reduction in lipid transport-related genes, and MI markedly reduces inflammatory markers, suggesting that it could prevent the progression from NAFLD to NASH. Furthermore, MI reduced fibrotic markers, proposing that it could also be considered as a treatment for advanced stages of NAFLD. Finally, this study also unraveled that the combination of these cofactors might have some additional beneficial effects on the amelioration of insulin resistance, and, therefore, in the development of NAFLD associated to HFHFr diets. To conclude, all these results suggest that supplementation with MI can be useful to improve obesity and an effective tool to treat NAFL and improve insulin resistance.

## Data Availability

Not applicable.

## Supplementary Materials

The following are available online at https://www.mdpi.com/article/10.3390/nu13103532/s1, Figure S1: Schematic diagram of the animal study; Figure S2a: Body weight before and after 4 weeks of MI supplementation; Figure S2b: Adiposity at the end of the supplementation calculated as the sum of as the white adipose depots (epididymal, retroperitoneal, inguinal and mesenteric); Figure S2c,d: Data of the solid and liquid food intake per week; Figure S3a: Fasting glycaemia; Figure S3b: Fasting insulinemia after MI supplementation.
