# Supplementary figures and images for: Supplementation with a Specific Combination of Metabolic Cofactors Ameliorates Non-Alcoholic Fatty Liver Disease, Hepatic Fibrosis, and Insulin Resistance in Mice
Source: Nutrients. 2021 Oct 9;13(10):3532. doi: 10.3390/nu13103532 (PMC8541294; doi:10.3390/nu13103532)

**Figure S1**

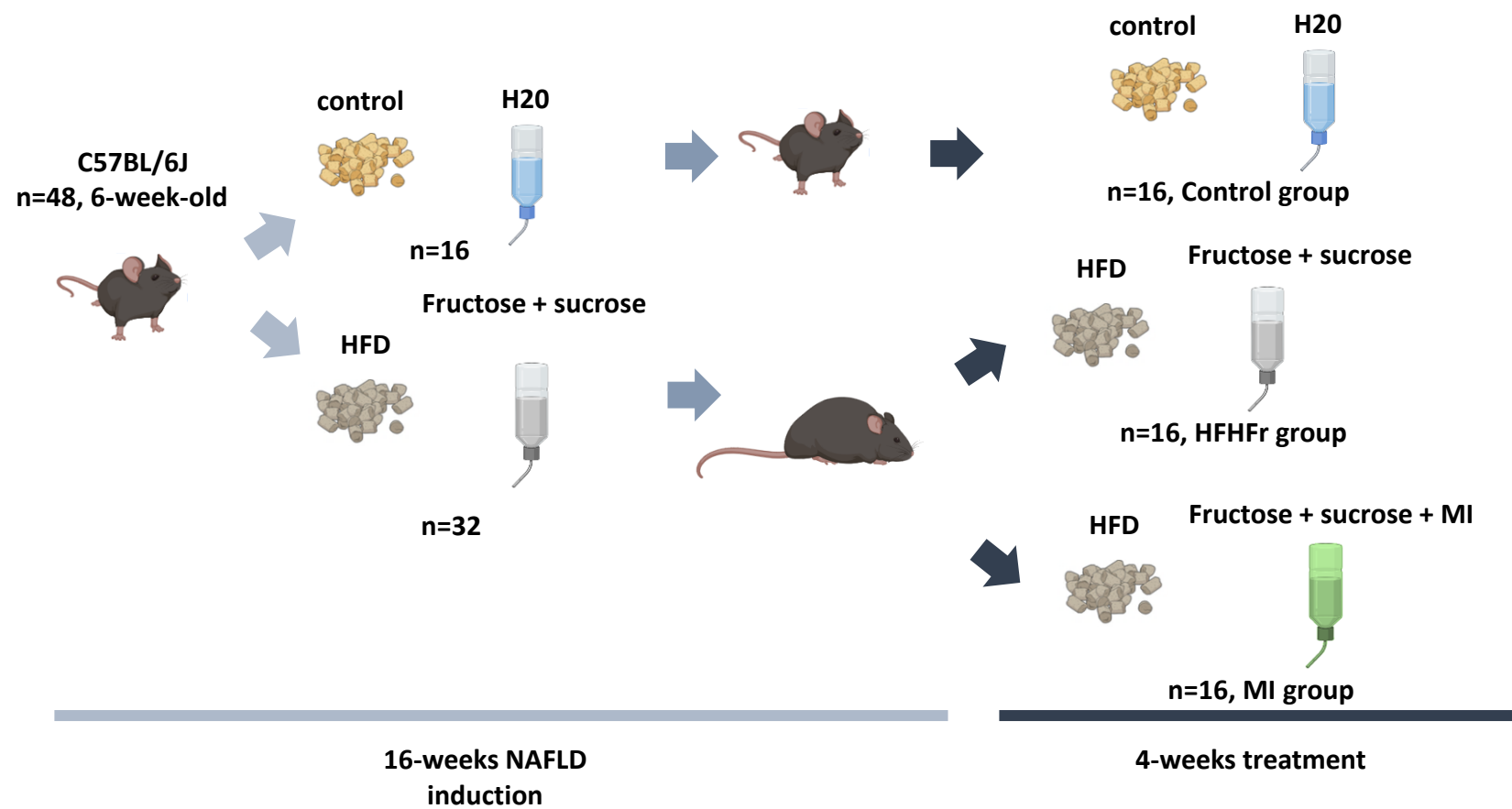

Figure S2

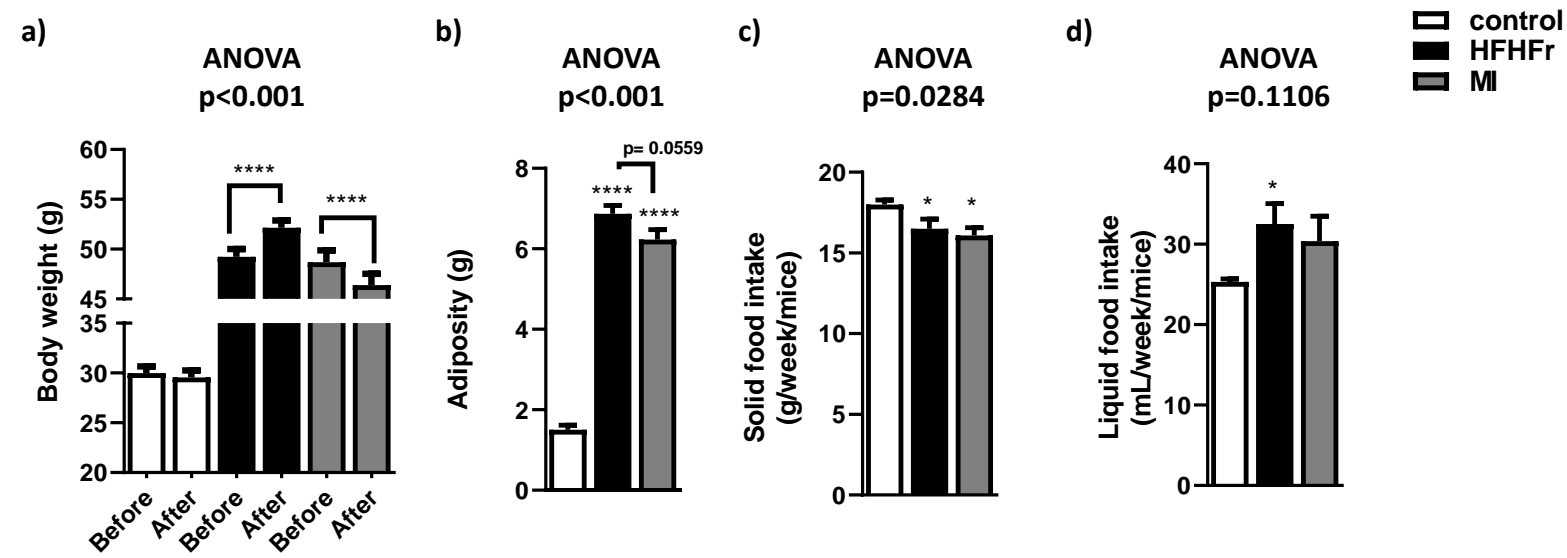

Figure S3

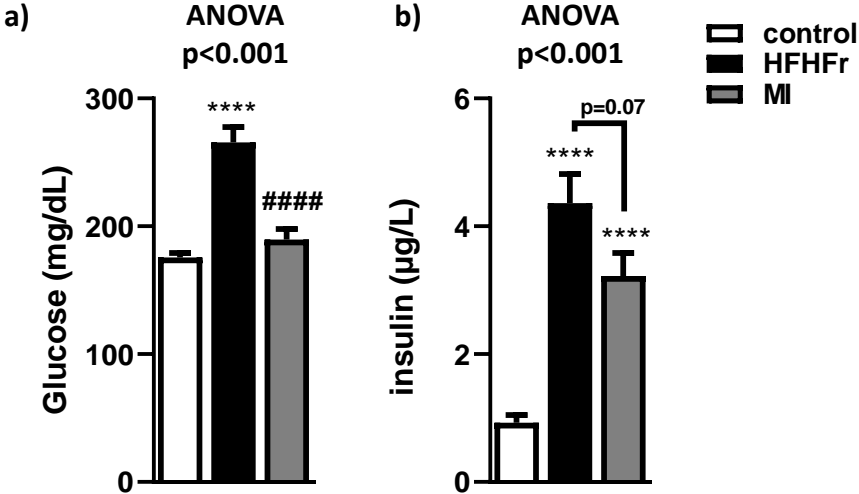

Supplement: Supplementary file 1 [file nutrients-13-03532-s001.zip › nutrients-1341424-supplementary.pdf]
